# Alcohol in Pneumonia

**Published:** 1905-07-15

**Authors:** 


### ALCOHOL IN PNEUMONIA.

This subject is still the battle-ground for rival therapeutic schools. Dr. Jas. Barr[1] considers that English practice is even yet under the influence of Todd, whilst in Scotland and Ireland the more moderate use of alcohol in febrile diseases can be traced to the influence of Graves, Stokes, and Sir Wm. Gairdner. He regards alcohol as of little value in pneumonia except as a soporific, and for this purpose prefers a light draught beer or stout. He gives this at nine or ten at night, and finds it especially useful in alcoholics and when the nervous system is irritable and there is much tremor. Again, in patients with small and irregular pulse and somewhat rigid vessels, a small amount of alcohol may do good, though better results are obtained from the use of atropine and nitroglycerine. Similarly, where there is flatulent distension of the abdomen a little brandy may give relief, but such antiseptics as salicylic acid and Dover's powder, tincture of iodine and glycerine, etc., are at least equally useful. In pneumonia complicated with delirium tremens, Dr. Barr allows two pints of beer or stout as a sedative, to be administered during the evening and night. Or a large dose of alcohol followed by a hypodermic injection of morphine and atropine is very good. Mechanical restraint or resistance carries the risk of syncope, but a few whiffs of chloroform may prove useful. In the selection of a hypnotic, attention should be paid to the condition of the pulse. If this is small but moderately firm, and the skin dry and not very moist, opium and antimony may be ordered; with lax and rather empty arteries, embarrassed respiration and very moist skin, Dr. Barr prefers a hypodermic injection of morphine and atropine, or morphine and strychnine. Lastly, alcohol has some virtue in the stage of convalescence. Here the heart is no longer stimulated by the heated blood, the blood-vessels have contracted, and the blood-pressure has risen. The enfeebled heart thus has to encounter increased peripheral resistance. In these circumstances a good port wine or a sound Burgundy will materially help to save the situation. Whilst Dr. Barr adopts the northern doctrine in reference to the use of alcohol in pneumonia, he evidently is far from accepting the teaching in reference to the use of opium which takes origin from the same source.

[1] Brit. Med. Jour., July 1, 1905.

